# Results of a planned interim toxicity analysis with trimodality therapy, including carboplatin AUC = 4, paclitaxel, 5-fluorouracil, amifostine, and radiation for locally advanced esophageal cancer: preliminary analyses and treatment recommendations from the North Central Cancer Treatment Group

**DOI:** 10.1186/1477-7800-1-9

**Published:** 2004-11-08

**Authors:** Aminah Jatoi, James Martenson, Michelle R Mahoney, Bradley S Lair, Jeffrey S Brindle, Frank Nichols, Normand Caron, Kendrith Rowland, Loren Tschetter, Steven Alberts

**Affiliations:** 1 Mayo Clinic, Rochester, Minnesota, USA; 2 Iowa Oncology Research Association Cancer Cooperative Oncology Program, Des Moines, Iowa, USA; 3 Altru Health Systems, Grand Forks, North Dakota, USA; 4 Toledo Community Hospital Cancer Cooperative Oncology Program, Toledo, Ohio, USA; 5 Sioux Community Cancer Consortium, Sioux Falls, South Dakota, USA

## Abstract

**Purpose:**

An aggressive trimodality approach from the Minnie Pearl Cancer Research Network [carboplatin AUC = 6, days 1 and 22; 5-fluorouracil 225 mg/m2 continuous infusion, days 1–42, paclitaxel 200 mg/m2, days 1 and 22; 45 Gy] has resulted in remarkable pathologic response rates but notable toxicity. This trial was designed to mitigate this toxicity by starting with a lower carboplatin dose, AUC = 4, and by adding subcutaneous amifostine.

**Methods:**

This phase II trial included patients with locally advanced, potentially resectable esophageal cancer. All were to receive the above regimen with modifications of carboplatin AUC = 4 and amifostine 500 mg subcutaneously before radiation. All were then to undergo an esophagectomy. A planned interim toxicity analysis after the first 10 patients was to determine whether the carboplatin dose should escalate to AUC = 6.

**Results:**

Ten patients were enrolled, and all required dose reductions/omissions during neoadjuvant therapy. One patient died from paclitaxel anaphylaxis. Six patients manifested a complete pathologic response.

**Conclusion:**

With this regimen, carboplatin AUC = 4 for patients with locally advanced esophageal cancer is appropriate.

## 

Combined modality treatment is often administered for locally advanced esophageal cancer [1,2]. Particularly noteworthy are data from Meluch and others from the Minnie Pearl Cancer Research Network [3]. These investigators published an innovative trial that examined neoadjuvant therapy that consisted of the following: carboplatin area under the curve (AUC) of 6 on days 1 and 22, paclitaxel 200 mg/m2 days 1 and 22, 5-fluorouracil 225 mg/m2 per day by continuous infusion days 1–42 along with radiation 45 Gy in 25 fractions. Yielding some of the most remarkable response rates observed in the treatment of esophageal cancer, this regimen resulted in a pathologic complete response rate of 46% within an initial cohort of 37 patients. Although long-term survival had not been reported, these data appear promising when one acknowledges that pathologic complete response predicts a longer survival and better prospect for cure for patients with this malignancy [4]. Moreover, recent preliminary data suggest that these response rates are reproducible [5,6].

However, this high complete response rate occurred at the expense of substantial treatment-related toxicity. During neoadjuvant therapy, grade 3 or worse leukopenia occurred in 65% of patients. Twenty-two percent of patients required hospitalization for neutropenic fevers. Grade 3 or worse esophagitis occurred in 31%. Over half of patients suffered weight loss of 10 pounds or more during neoadjuvant therapy. Finally, although there were no deaths during the administration of neoadjuvant therapy, the postoperative death rate was 9%.

These toxicity data, coupled with these high response rates, underscore the need to test such novel, aggressive treatments in conjunction with methods to mitigate toxicity. This report describes the planned interim toxicity analysis from an ongoing North Central Cancer Treatment Group (NCCTG) trial that was undertaken with this goal in mind. The NCCTG trial employed two modifications to the Minnie Pearl Regimen in an attempt to mitigate toxicity: 1) carboplatin dosing was reduced from AUC = 6 to AUC = 4 in the first 10 patients with the possibility of dose escalation thereafter; 2) amifostine 500 mg subcutaneously to be given before radiation, as prompted by data from Koukourakis and others [7] as well as by other subsequent reports [8]. An interim analysis of toxicity in this NCCTG trial was planned after enrollment of the first 10 patients to decide whether to continue this trial and, if so, whether to escalate the carboplatin to AUC = 6. The results of this interim toxicity analysis are presented below.

## Methods

### Overview

This trial was initiated and conducted within the NCCTG. The Institutional Review Boards at each site approved the protocol before patient enrollment, and all patients provided signed informed consent at the time of enrollment.

### Eligibility and Exclusion Criteria

Eligibility criteria consisted of the following: 1) age ≥ 18 years; 2) histologic or cytologic evidence of squamous cell carcinoma or adenocarcinoma of the esophagus; 3) surgically resectable tumor, as deemed by a surgeon; 4) Eastern Cooperative Oncology Group (ECOG) performance score of 0–2; 5) anticipated life expectancy of ≥ 12 weeks; 7) the laboratory parameters < 14 days prior to registration of absolute neutrophil count ≥ 1.5 × 10^9^/L, platelet count ≥ 100 × 10^9^/L, total bilirubin ≤ 1.5 times the upper limit of normal, serum creatinine ≤ 1.5 times the upper limit of normal, asparatate aminotransferase ≤ three times the upper limit of normal.

Exclusion criteria consisted of the following: 1) uncontrolled infection; 2) prior chemotherapy for esophageal cancer; 3) pregnancy or unwillingness to utilize contraception if pregnancy was a possibility; 4) New York Heart Association classification III or IV; 5) other severe underlying illness that, in the opinion of the treating oncologist, would make the patient inappropriate for study entry; 6) prior radiation that would overlap anticipated radiation fields; 7) antihypertensive or diuretic medications that could not be safely discontinued, if necessary, for several days during study treatment.

### Treatment Regimen

The neoadjuvant treatment regimen consisted of both chemotherapy and radiation. Chemotherapy consisted of carboplatin with an area under the curve (AUC) of 4 to be given intravenously on days 1 and 22, paclitaxel 200 mg/m2 to be given intravenously on days 1 and 22, and 5-fluorouracil 225 mg/m2/day to be given by continuous intravenous infusion on days 1 through 42. In addition, amifostine was to be administered as a 500 mg flat dose subcutaneously immediately before each radiation treatment.

For evaluation purposes, a treatment "cycle" is defined by the administration of carboplatin and paclitaxel, where the initiation of these agents heralded the start of a new chemotherapy "cycle." In effect, cycle 1 occurred during days 1–21 of the treatment, and cycle 2 between days 22–42.

The protocol was written to allow for a dose increase of carboplatin to an AUC of 6 in the event that a planned interim toxicity analysis after the first 10 patients deemed this increase could be undertaken safely.

Radiation was prescribed at a total dose of 4500 centigray. Each fraction size was 180 centigray, and a total of 25 fractions were to be given.

Chemotherapy dose reductions were initiated based on toxicity. As determined by the National Cancer Institute's Common Toxicity Criteria (NCI CTC), version 2, Grade 3 or worse stomatitis, esophagitis, or diarrhea prompted holding 5-fluorouracil until symptoms were grade 2 or better. If treatment was held for diarrhea, the protocol called for resuming the continuous infusion 5-fluorouracil at 80% of the prior dose. 5-Fluorouracil that was held was not to be made up. Severe myelosuppression on weekly blood counts (absolute neutrophil count ≥ 0.5 × 10^9^/L for greater than 2 days and/or platelet count ≤ 25 × 10^9^/L) prompted a 25% dose reduction of both paclitaxel and carboplatin on day 22. Similarly, on day 22, the protocol mandated that paclitaxel and carboplatin be held until the absolute neutrophil count was ≥ 1.5 × 10^9^/L and the platelet count ≥ 100 × 10^9^/L. For grade 3 or worse esophagitis, the paclitaxel and carboplatin were held until the esophagitis resolved to grade 1 or less. Paclitaxel was also held for grade 3 or worse neuropathy. Re-treatment was permitted with a 30% dose reduction in the event the neuropathy resolved to grade 2 or better. For any grade 3 or 4 event attributable to amifostine, the amifostine was to be decreased to 300 mg and subsequently discontinued if the event recurred at the lower dose. Finally, radiation was to be held for myelosuppression (absolute neutrophil count < 1.0 × 10^9^/L and/or the platelet count < 50 × 10^9^/L) or for grade 4 esophagitis.

Aggressive supportive care measures were recommended throughout the neoadjuvant portion of the regimen. These measures included, but were not limited to, premedication with corticosteroids prior to paclitaxel, use of antiemetics before and during chemotherapy and before amifostine, and nutrition and hydration support, as needed.

An esophagectomy was to be performed within 4–8 weeks after completion of radiation for all patients still deemed to be operative candidates.

The protocol also included an optional, additional two cycles of post-operative chemotherapy with paclitaxel and carboplatin with dosing, for the most part, left to the discretion of the treating oncologist.

### Pretreatment and Follow Up Evaluations

All patients underwent a history and physical examination within 21 days of trial registration. Other testing was performed within this time frame as well and included a complete hemogram, chemistry profile, computed tomography scan or magnetic resonance imaging of the chest and abdomen, chest radiograph, electrocardiogram, and an esophagoscopy. A bone scan was required if the alkaline phosphatase was two times the institution's upper limit of normal, and a bronchoscopy was required if the primary tumor was adjacent to the trachea or left main stem bronchus or if the patient was experiencing respiratory symptoms. Other testing was optional and included endoscopic ultrasound of the upper gastrointestinal tract and positron emission tomography scanning.

All patients were monitored throughout the period of radiation and chemotherapy administration with a weekly history and physical examination and weekly hemograms. History, physical examination, hemogram and chemistry profiles were mandated before days 1 and 22 of chemotherapy.

Tumor assessments were performed two weeks prior to surgery, and RECIST criteria, as recommended by the NCI , were used to determine tumor response [9]. Additionally pathological response was assessed post-operatively.

Other testing, such as quality of life assessment and genotyping, were also obtained but will be reported at a later date.

### Statistical Analyses

The purpose of this study was to assess the safety of this treatment regimen for patients with locally advanced esophageal cancer. The protocol called for a three-stage phase II study design with a safety analysis in the first 10 patients. The first 10 patients received treatment, as described above, with a carboplatin AUC = 4. It was decided *a priori *that if more than two of the initial patients developed grade 4 or 5 non-hematologic adverse events during neoadjuvant therapy or died within 15 days of surgery, then the study would not permit a carboplatin dose increase to an AUC = 6.

All data on toxicity and response are presented descriptively in this initial 10-patient report on the first phase of this trial. Summary statistics, including means and median values, frequency tables, and graphical methods were used to describe the distributions of drug administration, clinical characteristics, and adverse event rates. Unless otherwise specified, all adverse event data are reported regardless of relationship to study treatment.

## Results

### Demographics

Ten patients were recruited from late April 2001 to early March 2002. Patient characteristics are listed in Table 1, which shows that the median age of the cohort was 63 years (range 49–81) and that nine patients had an Eastern Cooperative Performance Score of 0 with one having a score of 1.

**Table 1:** 

**Characteristic**	**N = 10**
Age Median, in years (range)	63 (49–81)
Male/Female	9/1
Performance Score (ECOG)	
0	9
1	1

### Drug Administration

During administration of neoadjuvant therapy, all 10 patients required a dose reduction or an omission of drug administration because of treatment-related toxicity (Table 2). Overall, nine patients received both cycles of neoadjuvant chemotherapy.

**Table 2:** 

**Patient**	Amifostine dose received (%)*	Neoadjuvant chemotherapy dose received (%) 1^st^cycle/2^nd^cycle*	**Disease Status**
1	31	81/75	CR**
2	7	71/57	CR
3	16	64/11	PR***
4	27	100/26	PR
5	73	100/100	CR
6	24	52/3	CR
7	63	62****	PR
8	87	86****	dead
9	7	91/28	CR
10	67	72/79	CR

*denotes the lowest percentage of administered drug for a cycle

** complete response (CR)

***partial response (PR)

****second cycle not give

### Toxicity

Severe adverse events during neoadjuvant therapy consisted of 1 death (paclitaxel-related allergic reaction); 15 grade 4 events (myelosuppression (11); mucositis (1); dyspnea (1); neutropenia (1); and cerebral vascular accident (1); and 55 grade 3 events of various types (Table 3). Six of eight patients experienced grade 3 or greater adverse events within 15 days of surgery, and four of these were grade 4 events. Some patients experienced more than one event. Specifically, two patients suffered acute respiratory distress syndrome; one patient thrombophlebitis; two patients dyspnea; one patient non-specific constitutional symptoms; one patient neutropenia; and one patient a dysrhythmia. Of the two patients with acute respiratory distress syndrome, one remains alive 6 months after surgery as of this report, and the other died roughly 3 months after surgery.

**Table 3:** Salient Grade 3 or Worse Adverse Events Attributed to Neoadjuvant Study Treatment

**ADVERSE EVENT**	**ABSOLUTE INCIDENCE**	**NUMBER OF PATIENTS WITH EVENT**
death (paclitaxel reaction)	1	1
neutropenia	11	9
leukopenia	12	10
febrile neutropenia	5	5
hypotension	4	3
vomiting	4	3
nausea	2	2
dehydration	2	2
diarrhea	2	2
dysphagia	6	6
electrolyte abnormalities	6	4
dyspnea	1	1
pain (non-specific)	1	1
rash	1	1
mucositis	1	1
fatigue	2	2
syncope	1	1
hypersensitivity	1	1
neuropathy	1	1
infection	1	1
melena	1	1

### Preliminary Response Data

A total of 9 patients underwent an esophagectomy, demonstrating 6 complete pathologic responses.

## Discussion

This report describes a planned interim toxicity analysis of the first 10 patients who were enrolled on a multi-institutional trial for patients with locally advanced esophageal cancer. This NCCTG trial tested a version of the Minnie Pearl Regimen with two main modifications: a drop in the carboplatin AUC dose from 4 to 6 and an addition of amifostine 500 mg subcutaneously (flat dose) prior to each dose of radiation. Preliminary analyses show that this modified regimen resulted in a substantial rate of severe toxicity: one death from a paclitaxel allergic reaction, several episodes of grade 4 neutropenia/leukopenia, one episode of grade 4 mucositis as well as several grade 3 events – all of which were attributable to the study regimen. At the same time, however, this regimen resulted in 6 of 10 patients manifesting a complete pathologic response. The results of this planned analysis led to the NCCTG's decision to reopen this trial with a carboplatin dose of AUC = 4. In effect, the results of this interim analysis suggest that oncologists who might choose to treat patients with the Minnie Pearl Regimen should consider treating with this lower carboplatin dose, especially should they choose to include amifostine for its purported cytoprotective effects.

In fact, a major modification of the Minnie Pearl Regimen in the NCCTG trial described here is the addition of subcutaneous amifostine. It remains unclear whether the addition of amifostine compounded or mitigated the toxicity observed in this trial. Certainly, the inclusion of amifostine into this regimen did not permit a dose escalation of carboplatin to an AUC of 6, as tested in the original Minnie Pearl Regimen. Although amifostine is considered cytoprotective, it also carries toxicity in its own right including nausea, vomiting, and hypotension. Preliminary data suggest that subcutaneous administration of amifostine might be better tolerated than intravenous administration, but these conclusions are not based on large, comparative studies. The fact that the amifostine, carboplatin, 5-fluorouracil, paclitaxel, and radiation were all given in combination as part of the phase II study presented here makes it impossible to sort out attribution as it pertains to an individual study agent, such as amifostine. Should the results of this trial continue to appear promising, a larger phase III trial would be necessary to provide a definitive answer on the contribution of subcutaneous amifostine to the efficacy and toxicity profile of this regimen.

In short, the preliminary toxicity assessment of this trial suggests that the Minnie Pearl Regimen should include a carboplatin dose with an AUC of 4 rather than 6, especially if subcutaneous amifostine is included as part of the regimen.
